# The Inflammatory–Dysplastic Spectrum in Oral Lichen Planus: A Study on Six Immunohistochemical Markers

**DOI:** 10.3390/diagnostics15192443

**Published:** 2025-09-25

**Authors:** Oana Mihaela Condurache Hrițcu, Victor-Vlad Costan, Ștefan Vasile Toader, Delia Gabriela Ciobanu Apostol, Carmen Solcan, Daciana Elena Brănișteanu, Mihaela Paula Toader

**Affiliations:** 1 Department of Surgicals, Faculty of Dental Medicine, Grigore T. Popa University of Medicine and Pharmacy, 700115 Iasi, Romania; oana.condurache-hritcu@umfiasi.ro (O.M.C.H.); victor.costan@umfiasi.ro (V.-V.C.); mihaela.toader@umfiasi.ro (M.P.T.); 2 Department of Physiopathology, Faculty of Dental Medicine, Grigore T. Popa University of Medicine and Pharmacy, 700115 Iasi, Romania; 3 Department of Morpho-Functional Sciences I, Faculty of Medicine, Grigore T. Popa University of Medicine and Pharmacy, 700115 Iasi, Romania; delia.ciobanu@umfiasi.ro; 4 Department of Molecular Biology, Histology and Embryology, Faculty of Veterinary Medicine, University of Agricultural Science and Veterinary Medicine Ion Ionescu de la Brad, 8, Mihail Sadoveanu Alley, 700489 Iasi, Romania; carmensolcan@yahoo.com; 5 Department of Dermatology, Faculty of Medicine, Grigore T. Popa University of Medicine and Pharmacy, 700115 Iasi, Romania; daciana.branisteanu@umfiasi.ro

**Keywords:** oral lichen planus, immunohistochemistry, IL-17, maspin, β-Catenin, TIMP-1, MMP-14, Syndecan-4, inflammation, dysplasia

## Abstract

**Background/Objective:** Oral lichen planus (OLP) is a chronic inflammatory, immune-mediated mucosal condition classified as a potentially malignant disorder due to its risk of progression to oral squamous cell carcinoma (OSCC). The molecular events linking chronic inflammation in OLP to epithelial dysplasia remain poorly defined. To evaluate the expression of six immunohistochemical markers: IL-17, Maspin, β-Catenin, TIMP-1, MMP-14 and Syndecan-4 in OLP specimens and to explore their association with clinicopathological features and early dysplastic changes. **Methods:** We conducted a retrospective, cross-sectional study including 63 cases of OLP and 20 healthy controls. Formalin-fixed, paraffin-embedded sections underwent immunohistochemical staining for the six markers. Semi-quantitative scoring of staining intensity and percentage of positive cells was performed independently by two blinded pathologists. **Results:** IL-17 was markedly upregulated in 82.5% of OLP lesions versus absence in controls, correlating strongly with inflammatory infiltrate intensity. β-Catenin exhibited cytoplasmic and nuclear accumulation in 88.9% of OLP samples, with nuclear localization significantly associated with moderate dysplasia. Syndecan-4 membrane expression was reduced in dysplastic lesions, while Maspin and TIMP-1 co-expression were more prevalent in non-dysplastic OLP. MMP-14 was weakly positive in 87.3% of OLP cases and correlated with neovascularization. **Conclusions:** Elevated IL-17 expression and nuclear localization of β-Catenin may contribute to the progression of OLP toward dysplastic transformation, with this pattern being most evident in the erosive subtype. These findings suggest that a combined immunohistochemical panel may support risk stratification in OLP, although validation in larger, prospective cohorts is warranted.

## 1. Introduction

Oral lichen planus (OLP) is a chronic, immune-mediated inflammatory disorder of the oral mucosa that affects approximately 1–2% of the general population, with a strong female predilection in middle age [1]. The World Health Organization recognizes OLP as an oral potentially malignant disorder (OPMD) because longitudinal studies report a pooled malignant transformation rate of about 1.1% (range 0.4–1.8%) over follow-up periods averaging six years [2,3,4]. Clinically, OLP presents in several distinct variants—reticular (marked by asymptomatic Wickham’s striae), atrophic/erosive (painful, ulcerated), plaque-like (mimicking leukoplakia), and rarely bullous—with atrophic and erosive forms conferring the highest risk of progression to OSCC [3,5,6].

The pathogenesis of OLP centers on an antigen-specific, CD8^+^ T-cell attack against basal keratinocytes, resulting in vacuolar degeneration, apoptotic cell death, and disruption of epithelial integrity, accompanied by a dense, band-like lymphocytic infiltrate in the superficial lamina propria [7,8]. Persistent inflammation in this microenvironment generates pro-inflammatory cytokines (notably IL-17), reactive oxygen species, and growth factors that can induce DNA damage, aberrant repair, and epithelial dysplasia [9,10]. However, histopathologic distinction between reactive atypia and true dysplasia in OLP is notoriously challenging due to overlapping morphological features and significant inter-observer variability [11,12], underscoring the need for objective molecular markers to improve diagnostic reproducibility and risk stratification.

Recent advances in molecular pathology have highlighted several immunohistochemical (IHC) markers involved in inflammation, epithelial adhesion, and extracellular matrix remodeling, which may clarify the inflammatory–dysplastic continuum in OLP. Interleukin-17 (IL-17), secreted by Th17 cells, sustains chronic mucosal inflammation and promotes angiogenesis and keratinocyte proliferation [13,14,15]. Syndecan-4, a transmembrane heparan-sulfate proteoglycan, regulates cell–matrix adhesion and cytoskeletal dynamics; its downregulation correlates with loss of epithelial polarity and increased invasiveness in premalignant lesions [16,17,18]. Maspin, a serpin family protein with tumor-suppressor activity, exhibits nuclear localization in differentiated epithelia and its loss associates with poor prognosis in head and neck cancers [19,20]. β-Catenin, a central mediator of Wnt signaling and cell–cell adhesion, accumulates in the cytoplasm and nucleus during early dysplasia and OSCC, driving transcription of oncogenic targets [21,22,23]. Matrix metalloproteinase-14 (MMP-14) degrades basement membrane components to facilitate invasion, while its inhibitor TIMP-1 maintains extracellular matrix homeostasis; disruption of their balance underpins early stromal remodeling and epithelial–mesenchymal transition (EMT) [24,25,26].

The primary objective of this study was to investigate the immunohistochemical expression of six biomarkers—IL-17, Maspin, β-Catenin, TIMP-1, MMP-14, and Syndecan-4—in oral lichen planus (OLP) compared with healthy oral mucosa. Specifically, we aimed to assess their association with the presence and severity of epithelial dysplasia and to determine whether particular expression patterns may serve as predictors of malignant risk.

## 2. Materials and Methods

### 2.1. Study Design and Specimen Collection

This retrospective observational study was based on biopsy specimens archived between January 2015 and January 2023 at the Departments of Anatomical Pathology of the “Sfântul Spiridon” Emergency Clinical Hospital. A total of 63 cases with histopathologically confirmed oral lichen planus (OLP) were included. All cases strictly met the modified WHO clinical and histological diagnostic criteria for OLP. Lesions attributable to lupus erythematosus were excluded using a combination of clinical and histopathological criteria: (a) absence of systemic or mucocutaneous features suggestive of lupus, (b) no prior history of autoimmune disease, and (c) lack of histopathological hallmarks typical of lupus erythematosus, such as basement membrane thickening, perivascular inflammatory infiltrates, and deep connective tissue involvement.

Patients with systemic inflammatory diseases were excluded on the basis of medical history, clinical records, and routine laboratory screening. At the time of biopsy, all participants underwent a standard laboratory panel, including complete blood count, C-reactive protein (CRP), and liver and renal function tests. Individuals with autoimmune conditions (e.g., lupus erythematosus, rheumatoid arthritis) or chronic infections were also excluded.

For the control group, perilesional specimens of clinically normal, non-inflamed oral mucosa were obtained during minor surgical procedures. Inclusion criteria comprised adults over 18 years of age with no chronic illnesses and a confirmed clinical and histopathological diagnosis of either OLP or normal mucosa. Exclusion criteria included current smoking or alcohol abuse, prior radiotherapy to the head and neck, immunosuppressive therapy, systemic inflammatory disease, or absence of histopathological confirmation of the clinical diagnosis (Table 1).

The study was approved by the Ethics Committee of the “Grigore T. Popa” University (approval no. 61/23 March 2021), and written informed consent was obtained from all patients.

### 2.2. Reagents and Antibodies

The primary antibodies used for immunohistochemical analysis are listed in Table 2.

### 2.3. Tissue Processing and Staining Protocols

For immunohistochemical analysis, 4-µm sections were first deparaffinized through three consecutive xylene baths (10 min each) and then rehydrated in descending ethanol series (100%, 95%, 70%; 10 min each). Antigen retrieval was carried out in citrate buffer (pH 6.0) at 97–99 °C for 15 min. Endogenous peroxidase activity was quenched and nonspecific protein binding blocked by incubating the slides for 20 min each in commercial Peroxidase Block and Protein Block solutions. Sections were then incubated overnight at 4 °C with primary antibodies directed against IL-17, Syndecan-4, Maspin, β-Catenin, MMP-14, and TIMP-1 at their optimized dilutions. After PBS washes, slides were treated for 10 min with a biotinylated secondary antibody, followed by a 60-min incubation with streptavidin–peroxidase. Chromogenic detection was achieved by applying DAB substrate (30 µL DAB chromogen in 1.5 mL buffer) for 1 min, producing a brown reaction product at antigen sites. Finally, sections were counterstained in Mayer’s hematoxylin (1 min), dehydrated through graded ethanols (10 min each), cleared in xylene (9 min), coverslipped, and examined on an Olympus BX40 (Olympus Corporation, Tokyo, Japan) microscope. Positive controls included breast and colon carcinoma tissues. Negative controls were obtained by omitting the primary antibody.

### 2.4. Evaluation of Immunohistochemical Staining

Immunoreactivity for all six markers (IL-17, Maspin, β-Catenin, TIMP-1, MMP-14, and Syndecan-4) was semi-quantitatively scored against resident tissue lymphocytes, using a four-tier system: Negative (–); Weak (+, 0–10% of positive cells); Moderate (++); and Strong (+++, intensity equivalent to lymphocytes). Two independent, board-certified pathologists—both co-authors of this study—performed blinded evaluations of at least 1000 epithelial cells per case across ten high-power fields (40×). Oral lesions were further classified by anatomical location and histological grade of dysplasia: 0 = none, 1 = mild, 2 = moderate, 3 = severe. Cellular localization was documented for each antibody: IL-17 and Syndecan-4 (cytoplasmic/membranous), Maspin (nuclear and/or cytoplasmic), β-Catenin (membranous, cytoplasmic, nuclear), and MMP-14 and TIMP-1 (cytoplasmic and membranous), with notation of focal versus diffuse staining. Normal mucosa served as the baseline control. Any inter-observer discrepancies exceeding 10% were resolved by joint review and consensus. No automated or digital image-analysis software was applied.

### 2.5. Statistical Analysis

Data were entered into Microsoft Excel (Microsoft Corp., Redmond, WA, USA) and analyzed using IBM SPSS Statistics for Windows, Version 26.0 (IBM Corp., Armonk, NY, USA). Statistical significance was defined as *p* < 0.05. Group means were compared using Student’s *t*-test (according to degrees of freedom), while frequency distributions were assessed with the Chi-squared (χ^2^) test, applying Yates’ correction when appropriate. Odds Ratios (OR) and Relative Risks (RR) were also calculated to evaluate the likelihood of disease occurrence in exposed versus unexposed groups.

## 3. Results

### 3.1. Clinicopathological Characteristics

Given that 95% of the cohort represented erosive OLP, the findings of this study primarily reflect this clinical subtype. The buccal mucosa was the most commonly involved site, followed by the lateral tongue border and gingiva, with many patients presenting lesions at more than one oral location (Table 3). The control group comprised 20 specimens of perilesional normal mucosa, obtained from non-inflamed tissue adjacent to the lesion during biopsy.

### 3.2. Histological and Dysplasia Grades

All OLP cases met the World Health Organization histological diagnostic criteria, exhibiting basal cell degeneration, saw-tooth rete ridges, and a band-like lymphocytic infiltrate. Epithelial dysplasia was present in a subset of lesions: 24 cases (38.1%) showed mild dysplasia, and 8 cases (12.7%) displayed moderate dysplasia. No dysplastic changes were observed in control tissues (Table 4).

### 3.3. Immunohistochemical Expression

#### 3.3.1. Interleukin-17 (IL-17)

**Figure 1:**
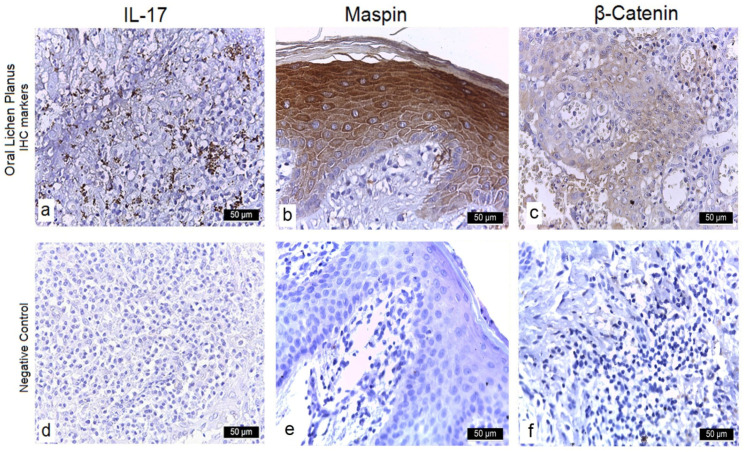
Immunohistochemical analysis. (**a**) Lichen planus—buccal mucosa, ×20 (IL-17): IL-17 immunostaining reveals an inflammatory lymphocytic infiltrate at the junction between the stratified squamous epithelium and underlying connective tissue, with degenerated basal keratinocytes. Strong IL-17 expression is present in lymphocytic aggregates and in keratinocytes of the basal layer. (**b**) Lichen planus—jugal mucosa, ×40 (maspin): Maspin immunostaining of a fragment of stratified squamous epithelium with hyperkeratosis over loose connective tissue shows collagen fibers, granulocytic inflammatory cells, isolated lymphocytes, and newly formed vessels with open lumens. Intense cytoplasmic positivity with occasional nuclear staining is observed in the epithelial cells. (**c**) Lichen planus—buccal mucosa, ×10 (β-catenin): β-Catenin immunostaining highlights superficial micro-erosions, hyperkeratosis, and loss of cellular cohesion in the deeper and granular layers of the stratified squamous epithelium, with an accompanying inflammatory infiltrate at the epithelium–lamina propria interface and within the superficial and deep lamina propria. Intense cytoplasmic staining and focal nuclear positivity are noted in the granular and clear layers, as well as superficially. (**d**) Normal buccal mucosa, ×20 (IL-17): IL-17 immunostaining of non-lesional stratified squamous epithelium overlying unremarkable lamina propria reveals occasional faint cytoplasmic staining in basal keratinocytes was observed, but below the threshold for positivity (<10% at 1+) and thus classified as negative. (**e**) Normal buccal mucosa, ×40 (maspin): Maspin immunostaining demonstrates weak, diffuse cytoplasmic positivity restricted to basal and parabasal epithelial layers, with no nuclear signal and absence of staining in the underlying connective tissue or vascular endothelium. (**f**) Normal buccal mucosa, ×10 (β-catenin): β-Catenin immunostaining highlights crisp membranous localization throughout all epithelial layers, preserving intercellular adhesion complexes and showing no cytoplasmic or nuclear accumulation in keratinocytes or stromal cells.

In the OLP cohort, 52 cases (82.5%) exhibited strong (+++) cytoplasmic and membranous IL-17 staining in basal and suprabasal layers, with no nuclear signal. These also showed intense (+++) inflammatory infiltrates and neovessel formation (+) in the lamina propria. The remaining 11 cases (17.5%) demonstrated moderate (++) IL-17 expression, moderate infiltrates (++), and presence of neovessels (+). All 20 control specimens were considered negative (–), as only occasional faint cytoplasmic staining in scattered basal keratinocytes was below the threshold (<10% cells at 1+). (Table 5) (Figure 1a,d).

#### 3.3.2. Maspin

Strong (+++) cytoplasmic and weak (+) nuclear Maspin labeling occurred in 58 OLP samples (92%), accompanied by moderate (++) inflammatory infiltrates and neovessels (+). The other 5 cases (8%) showed moderate (++) cytoplasmic and weak (+) nuclear staining, with moderate (++) infiltrates and no neovascularization (–). Among controls, 19/20 were negative (–) in both compartments; one showed weak (+) cytoplasmic positivity without stromal inflammation (Table 5) (Figure 1b,e).

#### 3.3.3. β-Catenin

Fifty-six OLP specimens (88.9%) displayed intense (+++) cytoplasmic and moderate (++) nuclear β-catenin staining, with intense (+++) infiltrates and no neovessels (–). The remaining 7 cases (11.1%) exhibited moderate (++) cytoplasmic and nuclear reactivity and moderate (++) infiltrates. In controls, β-Catenin remained strictly membranous in basal layers, with no cytoplasmic or nuclear signal (Table 5) (Figure 1c,f).

#### 3.3.4. TIMP-1

**Figure 2:**
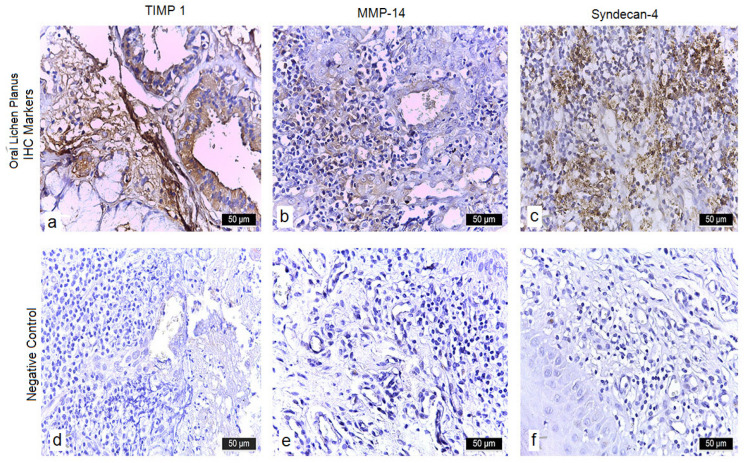
Immunohistochemical analysis. (**a**) Jugal mucosa—lichen planus, ×40 (TIMP-1): TIMP-1 staining of a tissue fragment containing glandular appendages, connective and adipose tissue with sparse lymphocytes demonstrated intense membranous labeling in the connective stroma, endothelial cells, adipocytes, and glandular structures, with prominent cytoplasmic positivity in all marked elements. (**b**) Jugal mucosa—lichen planus, ×20 (MMP-14): MMP-14 immunostaining in a hypercellular stroma rich in lymphocytes, plasma cells, and numerous thin-walled vessels with fibrillar connective material showed weak endothelial and membranous labeling, with faint cytoplasmic positivity observed in both lymphocytes and plasma cells. (**c**) Jugal mucosa—lichen planus, ×40 (Syndecan-4): Syndecan-4 staining highlighted the stratified squamous epithelium with an undulating basement membrane, intraepithelial micro-dehiscences forming small clear vacuoles, and superficial erosions overlying a loose, edematous lamina propria with abundant lymphocytic infiltrate. Intense membranous, cytoplasmic, interstitial, and endothelial membrane immunoreactivity was noted throughout the lesion. (**d**) Normal buccal mucosa, ×20 (TIMP-1): TIMP-1 immunostaining of non-lesional stratified squamous epithelium with intact parakeratotic layer overlying unremarkable lamina propria shows only faint, diffuse cytoplasmic positivity in scattered basal keratinocytes, without any stromal or inflammatory cell labeling. (**e**) Normal buccal mucosa, ×20 (MMP-14): MMP-14 immunostaining demonstrates absent to minimal cytoplasmic reactivity in all epithelial layers and underlying fibroblasts, with preservation of a continuous basement membrane and no perivascular or stromal expression. (**f**) Normal buccal mucosa, ×40 (syndecan-4): Syndecan-4 immunostaining highlights weak, uniform membranous and focal cytoplasmic localization in basal and parabasal keratinocytes, with no detectable staining in the spinous or superficial layers and absence of signal in the underlying connective tissue.

In 49 OLP cases (77.8%), TIMP-1 showed strong (+++) membranous and moderate (++) cytoplasmic expression, coupled with weak (+) infiltrates and absence of neovascularization (–). Fourteen cases (22.2%) had moderate (++) membranous and weak (+) cytoplasmic staining with similar stromal features. Control tissues exhibited only faint (+) diffuse cytoplasmic positivity in scattered basal keratinocytes, without stromal or inflammatory cell labeling. (Table 5) (Figure 2a,d). When stratified by subtype, erosive OLP cases (*n* = 60) exhibited the same overall patterns of IL-17 and β-Catenin upregulation as the full cohort, while the three non-erosive cases showed weaker expression levels across markers. Due to the small number of non-erosive cases, no reliable statistical comparison could be performed.

#### 3.3.5. MMP-14

Weak (+) cytoplasmic and membranous MMP-14 positivity was seen in 55 OLP samples (87.3%), alongside intense (+++) inflammatory infiltrates and neovessels (+). The remaining 8 cases (12.7%) had weak (+) cytoplasmic expression, moderate (++) infiltrates, and neovascularization (+). Control specimens were uniformly negative (–) in both epithelium and stroma (Table 5) (Figure 2b,e).

#### 3.3.6. Syndecan-4

Syndecan-4 exhibited intense (+++) membranous and cytoplasmic staining in 51 erosive OLP cases (81%), with intense (+++) infiltrates and no neovessels (–). Twelve cases (19%) showed moderate (++) expression and moderate (+) infiltrates without neovascularization. In controls, only weak (+) membranous labeling was noted in the basal layer, with no cytoplasmic signal (Table 5) (Figure 2c,f).

### 3.4. Statistical Correlations

- β-Catenin and Dysplasia: Nuclear localization of β-catenin was observed predominantly in moderately dysplastic lesions (12.7% of cases), whereas it was uncommon in non-dysplastic cases (49.2% of the cohort) (χ^2^ test, *p* < 0.05).
- MMP-14 and Neovascularization: Positive MMP-14 expression showed a significant association with neovascularization in the lamina propria (χ^2^ test, *p* = 0.03), suggesting its role in early stromal remodeling.
- Syndecan-4 Loss and Dysplasia: Reduced Syndecan-4 expression was significantly correlated with the presence of epithelial dysplasia (50.8% of OLP cases) compared with non-dysplastic mucosa (49.2%) (χ^2^ test, *p* < 0.05), underscoring its role in maintaining epithelial polarity.

## 4. Discussion

### 4.1. Interpretation of Findings

This study investigated six immunohistochemical markers—IL-17, Maspin, β-Catenin, TIMP-1, MMP-14 and Syndecan-4—in erosive oral lichen planus (OLP) versus normal mucosa, aiming to clarify their roles in the inflammatory–dysplastic continuum of high-risk OLP. Our results implicate these proteins in chronic inflammation, epithelial polarity loss, basement membrane remodeling, and angiogenesis.

Intense cytoplasmic and membranous IL-17 expression in over 80% of OLP cases confirms Th17-mediated inflammation as a central pathogenic driver [27,28]. This finding aligns with prior reports of elevated IL-17 in OLP tissue and saliva, linked to epithelial proliferation and stromal activation [9,10]. Moreover, IL-17 fosters tumor-associated inflammation by promoting cytokine cascades, matrix remodeling, and neovascularization [29].

Syndecan-4 was strongly positive at the membrane and in the cytoplasm of most erosive OLP specimens [16,30]. Its downregulation has been tied to dysplasia and poorer outcomes in head and neck squamous cell carcinoma [16,31], and syndecans have been posited as early modulators of epithelial disorganization in premalignant oral lesions [17,31].

Maspin exhibited high cytoplasmic staining with weaker nuclear localization in OLP lesions [32], reflecting its dual tumor-suppressor and apoptosis-regulatory functions [19,20]. Nuclear maspin correlates with reduced invasive potential and favorable prognosis in head and neck cancers [19,33], and our observation of pronounced cytoplasmic maspin suggests an early defensive response in non-dysplastic OLP [33].

β-Catenin shifted from a strictly membranous pattern in controls to cytoplasmic and nuclear localization in OLP, especially in dysplastic samples [21,34]. Such redistribution signifies Wnt pathway activation and is associated with early carcinogenic signaling [34,35]. Nuclear β-Catenin accumulation has been repeatedly linked to loss of epithelial cohesion and neoplastic transformation in oral mucosa [22,34].

TIMP-1 was robustly expressed in both OLP and normal tissues, though dysplastic lesions showed modestly reduced cytoplasmic intensity [26,36]. Its co-expression with maspin in non-dysplastic OLP suggests a protective phenotype [37], whereas diminished TIMP-1 levels have been connected to extracellular matrix breakdown and tumor progression [26,36].

MMP-14 was weakly positive in OLP biopsies but absent in controls [38,39]. Its presence correlated with neovascularization, underscoring its role in stromal remodeling and angiogenesis during early epithelial–mesenchymal transition [38,40]. Even low MMP-14 activity can activate MMP-2 and degrade basement membrane, facilitating invasion [24,40].

### 4.2. Integration with Pathogenetic Mechanisms

These data reinforce OLP as a dynamic inflammatory–dysplastic spectrum: IL-17 and Syndecan-4 denote inflammatory and structural microenvironment changes; Maspin and β-Catenin reflect epithelial polarity and regulatory shifts; and MMP-14/TIMP-1 illustrate opposing forces in extracellular matrix remodeling. Together, they substantiate an inflammatory-driven dysplastic interface that may elude routine histology.

### 4.3. Comparison with Existing Literature

Our findings concur with biomarker studies in OLP and other OPMDs: elevated IL-17 in tissue and saliva [9,10,41], β-Catenin nuclear translocation as a dysplasia predictor [21,22] and Maspin/TIMP-1 alterations linked to progression in oral potentially malignant disorders and OSCC [32,36]. The innovation here lies in concurrently profiling all six markers within a single erosive OLP cohort, highlighting their coordinated dysregulation.

Several recent reports strengthen these observations. Larsen et al. [41] demonstrated distinct systemic cytokine signatures in OLP and lichenoid lesions, supporting the role of IL-17 dysregulation in the disease spectrum. Beyond inflammatory conditions, Jiang et al. [42] showed that IL-17A directly promotes metastasis in tongue squamous cell carcinoma via the miR-23b/versican axis, reinforcing its dual role in both inflammation and malignant progression. Our results align with those of Ladjevac et al. [43], who showed that IL-17 drives OSCC pathogenesis by sustaining inflammation, promoting angiogenesis, and enabling immune escape, supporting its role as an early marker of malignant progression.

β-Catenin alterations also appear central. Saleh et al. [44] confirmed significantly higher nuclear β-catenin in dysplastic OLP compared to non-dysplastic lesions, aligning with our data. In a previous study from our group, Condurache Hrițcu et al. [20] reported coordinated expression shifts of Maspin, β-catenin, and MMP-14 in OPMDs and OSCC, consistent with our current multi-marker approach.

Matrix remodeling markers further connect OLP to malignant potential. Nosratzehi et al. [45] found salivary increases in multiple MMPs (MMP-1, -2, -3, -13) in OLP and OSCC, while Zhang et al. [46] highlighted stage-dependent alterations of collagen IV, gelatinases, and TIMP-1 in oral leukoplakia. These findings corroborate our observation that proteolytic imbalance and TIMP-1 dysregulation may be subtype- or stage-specific rather than uniform.

Taken together, these studies emphasize that the inflammatory, proteolytic, and tumor-suppressive pathways explored in our erosive OLP cohort are not isolated but reflect a broader pathogenic continuum across OPMDs and OSCC.

### 4.4. Clinical Implications

This multi-marker IHC profile could inform risk stratification: IL-17 and nuclear β-Catenin may flag lesions at greater risk of dysplastic shift, whereas preserved Maspin and TIMP-1 might indicate epithelial stability. Incorporating these biomarkers into digital pathology workflows could enhance diagnostic reproducibility and guide personalized surveillance or intervention strategies [47,48].

### 4.5. Limitations

The present study has several limitations. First, the relatively small cohort size reduces statistical power and may limit the robustness of subgroup analyses. Second, because 95% of the included cases were of the erosive subtype, the findings cannot be fully generalized to other OLP variants such as reticular or plaque-like forms. Third, immunohistochemical assessment relied on semi-quantitative scoring by pathologists without the use of digital image analysis, which may introduce observer bias. Finally, the retrospective, cross-sectional design precludes causal inference, restricting interpretation to associations rather than predictive or mechanistic conclusions.

### 4.6. Future Directions

Prospective, multicenter studies with integrated transcriptomic and genomic profiling are needed to validate these markers. Advanced models—such as 3D organotypic cultures or ex vivo OLP mucosa—could deepen mechanistic insights. Ultimately, a standardized, multiplex IHC panel may become a cornerstone for individualized OPMD management.

## 5. Conclusions

This study demonstrates that oral lichen planus embodies a dynamic inflammatory–dysplastic spectrum, in which chronic immune activation and early molecular alterations converge to promote malignant potential. Our immunohistochemical panel revealed that elevated IL-17 and nuclear translocation of β-Catenin are associated with higher dysplastic risk, while preserved Maspin and TIMP-1 expression denote a more stable epithelial phenotype. Syndecan-4 loss and MMP-14 upregulation further underscore the roles of epithelial polarity disruption and stromal remodeling in OLP pathogenesis. Given the retrospective, cross-sectional design, causal relationships cannot be inferred and the findings should be interpreted as associative.

By integrating these six markers into a single cohort analysis, we provide an objective framework for distinguishing high-risk OLP subtypes that may benefit from intensified surveillance or adjunctive therapies. Although retrospective in design and limited by semi-quantitative scoring, our findings warrant prospective validation using digital image analysis and longitudinal follow-up to confirm their predictive value. Ultimately, a standardized multi-marker IHC panel could enhance diagnostic reproducibility, inform personalized risk stratification, and guide clinical management of patients with OLP.

## Figures and Tables

**Table 1:** Characteristics of the study group (OLP) and control group.

Characteristic	OLP Group (*n* = 63)	Control Group (*n* = 20)
Timeframe	January 2015–January 2023	January 2015–January 2023
Source	Archived biopsy specimens from the Department of Anatomical Pathology at “Sfântul Spiridon” Emergency Clinical Hospital	Normal oral mucosa obtained during minor surgical procedures (e.g., third molar extraction)
Inclusion Criteria	Age > 18 years; no other chronic illness; histopathologically confirmed OLP (modified WHO criteria)	Age > 18 years; no other chronic illness; clinically and histologically normal mucosa (no inflammation or dysplasia)
Exclusion Criteria	Current smokers or alcohol abuse; prior head and neck radiotherapy; immunosuppressive therapy; systemic inflammatory diseases; lack of histopathological confirmation;	Current smokers or alcohol abuse; prior head and neck radiotherapy; immunosuppressive therapy; systemic inflammatory diseases; lack of histopathological confirmation;

**Table 2:** Reagents and Antibodies.

Marker	Clone	Host	Dilution	Supplier	Catalog No.
IL-17	Polyclonal	Rabbit	1:100	Biorbyt, Cambridge, UK	ORB13500
β-Catenin	SAB4500543	Rabbit	1:50	Sigma-Aldrich, St. Louis, MO, USA	SAB4500543-100UG
Maspin	Clone C-8	Mouse	1:100	Santa Cruz Biotechnology, Dallas, TX, USA	SC-271694
MMP-14	SAB4501901	Rabbit	1:100	Sigma-Aldrich, St. Louis, MO, USA	SAB4501901-100UG
Syndecan-4	SAB4502721	Rabbit	1:100	Sigma-Aldrich, St. Louis, MO, USA	SAB4502721-100UG
TIMP-1	HPA053417	Rabbit	1:100	Atlas Antibodies, Bromma, Sweden	HPA053417-100UL

**Table 3:** Distribution of Affected Sites by OLP.

Affected Site	Number of Cases (*n* = 63)	Percentage (%)
Buccal mucosa	56	89.2
Lateral border of the tongue	37	58.6
Gingiva	11	16.7
Labial mucosa (lips)	11	16.7

**Table 4:** Comparison of Histopathological Features Between OLP and Control Groups.

Histopathological Feature	OLP Group (*n* = 63)	Control Group (*n* = 20)
Basal cell degeneration	Present in 63 (100%)	Absent
Saw-tooth rete ridges	Present in 63 (100%)	Absent
Band-like lymphocytic infiltrate	Present in 63 (100%)	Absent
Epithelial dysplasia—none	31 (49.2%)	20 (100%)
Epithelial dysplasia—mild	24 (38.1%)	0 (0%)
Epithelial dysplasia—moderate	8 (12.7%)	0 (0%)

**Table 5:** Immunohistochemical Expression of IL-17, Syndecan-4, Maspin, β-Catenin, MMP-14, and TIMP-1 in OLP versus control.

Marker	Localization	OLP Group				Control Group			
		(0)	(+)	(++)	(+++)	(0)	(+)	(++)	(+++)
IL-17	Cytoplasmic/Membranous	0 (0.0%)	11 (17.5%)	0 (0.0%)	52 (82.5%)	20 (100.0%)	0 (0.0%)	0 (0.0%)	0 (0.0%)
Maspin	Cytoplasmic/Nuclear	0 (0.0%)	0 (0.0%)	5 (7.9%)	58 (92.1%)	19 (95.0%)	1(5.0%)	0 (0.0%)	0(0.0%)
β-Catenin	Cytoplasmic/Nuclear	0 (0.0%)	0 (0.0%)	7 (11.1%)	56 (88.9%)	20 (100.0%)	0(0.0%)	0 (0.0%)	0 (0.0%)
TIMP-1	Membranous/Cytoplasmic	0 (0.0%)	0 (0.0%)	14 (22.2%)	49 (77.8%)	0 (0.0%)	20 (100.0%)	0 (0.0%)	0 (0.0%)
MMP-14	Cytoplasmic/Membranous	0 (0.0%)	63 (100%)	0 (0.0%)	0 (0.0%)	20 (100.0%)	0 (0.0%)	0 (0.0%)	0 (0.0%)
Syndecan-4	Membranous	0 (0.0%)	0 (0.0%)	12 (19.0%)	51 (81.0%)	0 (0.0%)	20 (100.0%)	0 (0.0%)	0 (0.0%)

Immunoreactivity was scored semi-quantitatively as + (weak), ++ (moderate), and +++ (strong, intensity equivalent to lymphocytes).

## Data Availability

The original contributions presented in this study are included in the article. Further inquiries can be directed to the corresponding author.

## Abbreviations

The following abbreviations are used in this manuscript

OLP	Oral lichen planus
EMT	Epithelial–mesenchymal transition
IHC	Immunohistochemical
OPMD	Oral potentially malignant disorder
OSCC	Oral squamous cell carcinoma
H&E	Hematoxylin and eosin
PBS	Phosphate-buffered saline
DAB	3,3′-Diaminobenzidine
Il-17	Interleukin-17
MMP-14	Matrix metalloproteinase-14
